# Genome-Wide Association Study for Body Conformation Traits in Kazakh Fat-Tailed Coarse-Wool Sheep

**DOI:** 10.3390/genes16091023

**Published:** 2025-08-29

**Authors:** Zhanerke Akhatayeva, Kairat Dossybayev, Altynay Kozhakhmet, Marina Yermekova, Tilek Kapassuly, Kanagat Yergali, Temirlan Kulboldin, Aibyn Torekhanov, Beibit Kulataev, Kairat Iskakov, Temirkhan Kenzhebaev, Xianyong Lan

**Affiliations:** 1 LPP “Kazakh Research Institute for Livestock and Fodder Production”, Almaty 050035, Kazakhstan; akhatayevazhanerke@163.com (Z.A.); altynaitg@gmail.com (A.K.); nurii_90@mail.ru (M.Y.); tilek.kapas@mail.ru (T.K.); ergaly.qanagat@gmail.com (K.Y.); k.temoha@gmail.com (T.K.); torehanov.aibyn@mail.ru (A.T.); bnar68@yandex.ru (B.K.); kairat11101988@mail.ru (K.I.); kterdesh@mail.ru (T.K.); 2 Institute of Grassland Research, Chinese Academy of Agricultural Sciences, Hohhot 010010, China; 3 RSE Institute of Genetics and Physiology SC MSHE RK, Almaty 050060, Kazakhstan; 4 Faculty of Biology and Biotechnology, Al-Farabi Kazakh National University, Almaty 050040, Kazakhstan; 5 College of Animal Science and Technology, Northwest A&F University, Yangling, Xianyang 712100, China; lanxianyong79@126.com

**Keywords:** sheep, GWAS, SNP, MAS, growth traits, association

## Abstract

Background: In Kazakhstan, there is a notable demand for fat-tail sheep breeds in both domestic and international markets, which has led to the prioritization of certain breeds for breeding purposes. Among the various sheep breeds raised in the desert and semi-desert regions of Kazakhstan, the Kazakh fat-tailed coarse-wool sheep is particularly valued for its production of high-quality mutton. Objective: This study aimed to identify genomic regions and candidate genes associated with body conformation traits in this breed using a genome-wide association study (GWAS). Methods: A GWAS was performed on 295 Kazakh fat-tailed coarse-wool using OvineSNP50 Genotyping BeadChip (Illumina, San Diego, CA, USA). Results: After quality control, 41,912 single-nucleotide polymorphisms (SNPs) remained for analysis. Several loci showed suggestive associations (*p* < 1 × 10^−5^) with growth traits. These included s23127.1 and OAR6_56152225.1 for live weight; s08490.1 for chest width; s22731.1 for oblique length; OAR10_1168444.1 for cannon bone circumference; and s58409.1 for both rump height and withers height. Candidate genes near these loci encompassed *VCAN*, *NEK1*, *NRG1*, *ADAM12*, *ERBB4*, *RUNX1T1*, and *PDGFD*. Conclusion: Thus, these genetic variations have the potential to serve as candidate markers for MAS targeting body conformation traits in Kazakh fat-tailed coarse-wool sheep.

## 1. Introduction

Currently, sheep breeding represents a vital sector within Kazakhstan’s agro–industrial complex, distinguished by the wide range of products it generates. Beyond providing meat and fat, it serves as a primary source of essential raw materials for the textile and leather industry, particularly wool and sheepskin [1]. Fat-tailed sheep are one of the most prevalent breeds in Kazakhstan, possessing several advantageous traits developed through centuries of natural and traditional selection in challenging environments [2]. A distinctive feature of this breed is the accumulation of fat at the base of the tail, forming a prominent structure known as a fat tail [3]. Fat-tailed sheep played a major role in the creation of new breeds and breed groups of fine-wool, semi-fine-wool and semi-coarse-wool sheep in Kazakhstan [4]. The Kazakh fat-tailed coarse-wool sheep was developed by traditional breeding and includes a number of breed types differing in productivity level and breeding areas [5].

Implementing selection strategies that focus on economically significant traits is crucial to enhance the productivity of sheep. One effective approach involves marker-assisted selection (MAS) using genotyping data [6]. Genome-wide association studies (GWAS) play an essential role in detecting single-nucleotide polymorphisms (SNPs) that can be targeted for genotyping through sequencing, as well as in uncovering new candidate genes linked to desirable productive traits [7]. However, genomic selection is not widely adopted in sheep breeds in Kazakhstan. The development of extensive genotyping databases can enhance the efficiency of predicting productive traits in livestock.

In sheep, most studies have focused on identifying genetic polymorphisms associated with meat traits, particularly in genes such as myostatin (*MSTN*), calpastatin (*CALP*), and insulin-like growth factor 1 (*IGF-1*) [8,9,10,11]. In previous research, SNPs in the *IGFBP6*, *FGF12*, *FTO*, ST7, *DTNBP1*, *SCD5*, *KYNU* genes that are related to growth traits were identified in Saryarka fat-tailed coarse-wool sheep [3]. Another study discovered a 168 bp insertion in the homeobox B13 (*HOXB13*) gene, which occurs at a high frequency in long-tailed sheep, using whole-genome sequencing [12]. Several GWAS identified genes (e.g., *ADGRL3*, *SPAST*, *TGFA*, *ELOVL2*, *ARAP2*, *IBN2*, *TPM*) are related to meat and carcass traits in sheep via Illumina OvineSNP50 BeadChip [13,14,15]. Moreover, two SNPs were identified as functional variants for growth traits in Hu sheep, with *CAPN6* emerging as a candidate gene showing differential expression in muscle tissues [16]. GWAS for birth weight identified significant SNP and candidate genes in Tan sheep [17]. Functional annotation identified 24 body weight-related genes, along with nine quantitative trait loci (QTLs) in Kazakh and Texel sheep [18].

However, there are currently no studies identifying potential markers associated with productive traits in Kazakh fat-tailed coarse-wool sheep breed. Therefore, this study aimed to reveal potential genes that are associated with growth traits via GWAS for further use in selection. The findings provide a theoretical foundation for future research into candidate genes that influence the body confirmation traits in Kazakh fat-tailed coarse-wool sheep.

## 2. Materials and Methods

### 2.1. Sample Collection and Data Record

**Figure 1:**
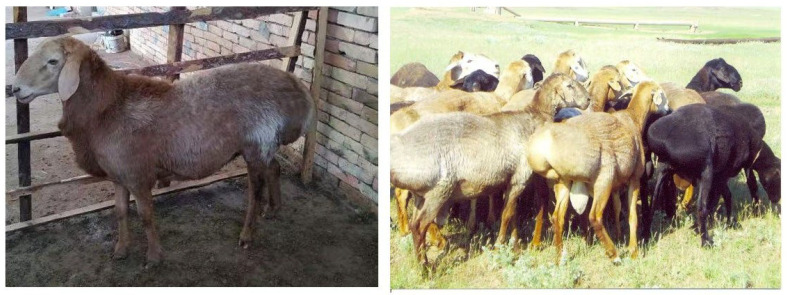
The ewe (**left**) and flock (**right**) of Kazakh fat-tailed coarse-wool sheep.

The Kazakh fat-tailed coarse-wool sheep sample comprised a total of 295 sheep (Figure 1). The animals were collected from a single farm. All sheep used in the analysis were healthy, physiologically mature adult females (2.5–3 years old), and they were maintained under similar environmental and feeding conditions. The experimental animals are raised in the South Kazakhstan region, which has a continental climate characterized by hot summers and mild to cold winters. The region consists of broad temperate steppe landscapes that gradually transition into foothills and mountainous areas. With increasing elevation, vegetation shifts from grass-dominated steppe communities to diverse subalpine and alpine meadows, offering high-quality forage during the summer months. This vertical zonation supported traditional transhumant pastoralism, in which flocks were seasonally moved between lowland and upland pastures to make optimal use of natural resources. Sheep were managed under extensive pastoral systems, grazing year-round on native steppe vegetation without supplemental concentrated feed. This approach aligns with the arid to semi-arid climatic conditions of the area, to which Kazakh fat-tailed coarse-wool sheep are well adapted, relying on continuous access to natural forage and water sources. Routine health care, including vaccination and parasite control, was administered according to standard regional veterinary practices. Housing was minimal and primarily used for protection during extreme weather conditions.

The LW (live weight), ChD (chest depth), ChW (chest width), ChG (chest girth), CBC (cannon bone circumference), OL (oblique length), HW (hip width), RH (rump height), and WH (withers height) were measured as described in a previous study [3]. Briefly, morphological parameters were measured following standard livestock measurement procedures. Height measurements (e.g., withers height, rump height) were obtained using a Lydin measuring stick (GENERICA, Moscow, Russia), while linear dimensions (e.g., oblique length, chest girth, chest depth) were recorded with a measuring tape. Body weight was measured using an electronic livestock scale.

### 2.2. DNA Extraction, Genotyping, and Quality Control

Genomic DNA was extracted from whole blood samples collected via jugular venipuncture using standard sterile procedures. The blood was stored in EDTA-containing tubes and processed using a GeneJET Genomic DNA Purification Kit from Thermo Scientific (Thermo Fisher Scientific, Waltham, MA, USA) DNA extraction kit following the manufacturer’s protocol. Briefly, the protocol involved enzymatic digestion with Proteinase K, followed by lysis, ethanol precipitation, and column-based purification with successive wash steps. DNA was eluted in 200 μL of Elution Buffer, yielding high-quality genomic DNA suitable for downstream applications. The quality of the extracted DNA was assessed using a NanoDrop One spectrophotometer and a Qubit Fluorometer (Thermo Fisher Scientific, Waltham, MA, USA). The DNA concentration was adjusted to 50–100 ng/µL for SNP genotyping.

The animals were genotyped by using the OvineSNP50 Genotyping BeadChip (Illumina Inc., San Diego, CA, USA). A quality control was performed using the Plink (V1.90) software [19]. SNPs were excluded from the analysis if the minor allele frequency (MAF) was less than 5%, the call rate was less than 98%, and genotype frequency deviated from Hardy–Weinberg Equilibrium (HWE) with a *p*-value lower than 0.001. A total of 49,363 SNPs were involved in quality control and 7451 SNPs removed after. As a result, a final set of 41,912 SNPs for 295 animals remained for further GWAS.

### 2.3. GWAS Analysis and Gene Annotation

The association analyses of traits were performed using a linear mixed model (LMM) in the GEMMA software (https://github.com/genetics-statistics/GEMMA, accessed on 10 June 2025). The following model was used for GWAS:

(1) y = Wα + xβ + u + ε; u~MVN_n_ (0, λτ ^− 1^ K), ε~MVN_n_ (0, λτ ^− 1^ I_n_)

where y denotes the target trait across n individuals and traits. W is the matrix of fixed effects, and α represents their corresponding coefficients, including the intercept. x refers to the SNP genotype, while β indicates the SNP’s effect size. u captures the random effects, and ε represents the residual errors. MVN_n_ signifies a multivariate normal distribution with n dimensions. τ^−1^ denotes the residual error variance, λ is the ratio between two parameters, and K is the kinship matrix, with I_n_ as the identity matrix [15].

A Manhattan plot was generated using R version 4.4.1, and the Bonferroni correction was applied to control the family-wise error rate. The suggestive association significance threshold was set at *p* < 1 × 10^−5^, while the genome-wide significance threshold was set at *p* < 5 × 10^−8^.

In addition, SNPs were annotated using the sheep reference genome (ARS-UI_Ramb_v2.0) assembly through Ensembl BioMart (https://mart.ensembl.org/index.html, accessed on 14 July 2025) to retrieve associated gene information.

### 2.4. Functional Enrichment Analysis of Candidate Genes

Functional enrichment analysis of the candidate genes associated with growth traits was conducted using the DAVID website (https://david.ncifcrf.gov, accessed on 16 July 2025) to identify the relevant Gene Ontology (GO) and Kyoto Encyclopedia of Genes and Genomes (KEGG) pathways.

## 3. Results

### 3.1. Statistics of Phenotype

Descriptive statistics of the phenotypic traits measured in 295 sheep are given in Table 1. Mean live weight (63.1 kg) shows moderate variability (CV = 6.2%), ranging from 52 to 73 kg, indicating relatively uniform body mass among individuals. Withers height (74.2 cm) and rump height (75.7 cm) have low coefficients of variation (CV = 4.8% and 4.6%, respectively), suggesting consistency in vertical body size. Chest width (25.7 cm) and hip width (20.1 cm) show higher variability (CV = 8.1% and 9.8%), indicating more individual differences in body breadth. Cannon bone circumference (9.2 cm) shows the highest variation (CV = 10.8%), suggesting a wider range in bone thickness among animals. Chest girth (101.5 cm) and oblique length (64.5 cm) show low to moderate variation, indicating stable torso size across individuals. Chest depth (35.4 cm) has a CV of 4.9%, reflecting consistency in thoracic development. Most traits exhibit low to moderate variability, suggesting a relatively homogeneous population. Diversity in some growth traits may indicate influence of management practices or genetic variation.

**Figure 2:**
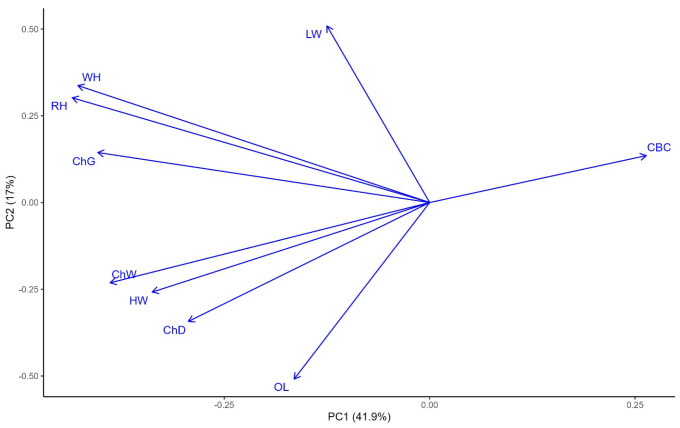
Principal component analysis of the body measurement traits in sheep. LW, live weight; ChD, chest depth; ChW, chest width; CBC, cannon bone circumference; OL, oblique length; ChG, chest girth; HW, hip width; RH, rump height; WH, withers height. The *x*-axis (PC1) and *y*-axis (PC2) represent the first and second principal components, which explain 41.9% and 17% of the total 59.6% phenotypic variance, respectively. Arrows indicate the direction and contribution of each trait to the PCs.

Principal component analysis (PCA) was conducted to explore the relationships among body conformation traits (see Figure 2). The first two principal components (PC1 and PC2) explained 41.9% and 17.7% of the total phenotypic variation, respectively. Traits such as CBC and LW were positively associated with PC1, while OL, ChW, and HW showed strong negative loadings, indicating a contrast between body thickness and length traits. PC2 primarily distinguished height-related traits such as WH, RH, ChG, ChW and ChD. The angle between vectors suggested strong positive correlations among WH and RH. These results highlight distinct trait groupings that contribute to overall body conformation in the studied population.

**Figure 3:**
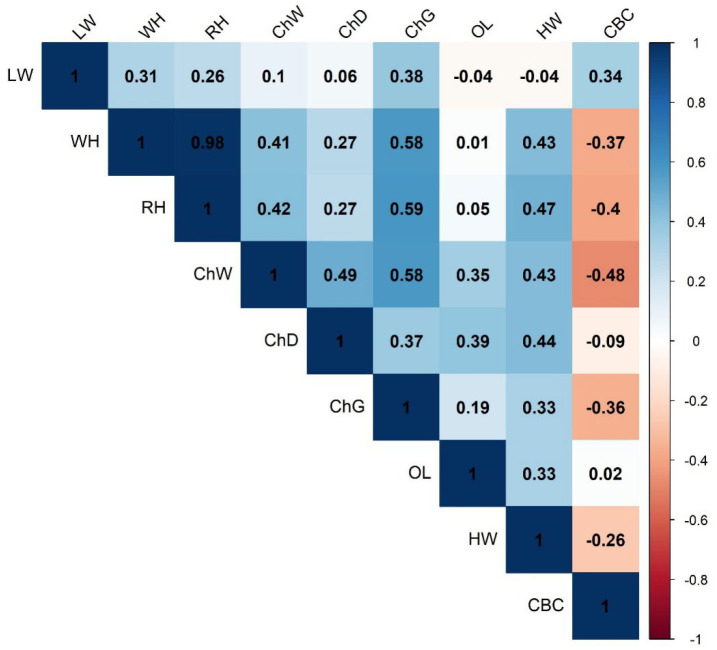
A correlation matrix heatmap for various body measurement traits. LW, live weight; ChD, chest depth; ChW, chest width; CBC, cannon bone circumference; OL, oblique length; ChG, chest girth; HW, hip width; RH, rump height; WH, withers height.

Figure 3 illustrates a correlation matrix heatmap for various body measurements. WH and RH (0.98) had very strong positive correlation; ChD and ChW (0.49), and LW and ChG (0.38) showed moderate correlations. Overall, most traits are positively correlated, especially height and weight traits. However, CBC tends to be negatively correlated with several other traits, suggesting it is more independent by the other variables.

### 3.2. Genome-Wide Association Study

**Figure 4:**
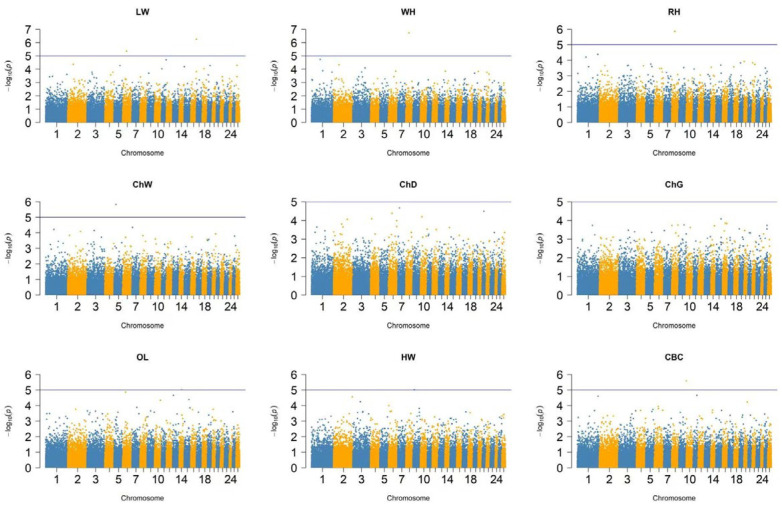
Manhattan plots of GWAS of Kazakh fat-tailed coarse-wool sheep: LW, live weight; WH, withers height; RH, rump height; ChW, chest width; ChD, chest depth; ChG, chest girth; OL, oblique length; HW, hip width; CBC, cannon bone circumference. The horizontal line represents the suggestive genome-wide significance threshold (*p* < 1 × 10^−5^).

Figure 4 presents the Manhattan plot illustrating body confirmation traits, based on a total of 41,912 SNPs spread across 26 chromosomes. Based on the calculated significance threshold, no SNPs reached genome-wide significance threshold (*p* < 5 × 10^−8^) in the tested sheep population. Additionally, Figure S1 displays the quantile–quantile (QQ) plots, which compare the distribution of the observed −log10 *p*-values of the SNPs to their expected distribution. The QQ plots indicate that the GWAS models are well-calibrated with minimal inflation, suggesting reliable control of population structure and false positives. As shown in Table S1, several SNPs were identified that reached genome-wide suggestive threshold *p* < 1 × 10^−5^).

Although no SNPs reached the genome-wide significance threshold (*p* < 5 × 10^−8^) in this study, several loci met the suggestive significance threshold (*p* < 1 × 10^−5^) and may represent genomic regions influencing body conformation traits in Kazakh fat-tailed coarse-wool sheep. For live weight, two suggestive SNPs, including s23127.1 on chromosome 16 (*p* = 5.47 × 10^−7^) and OAR6_56152225.1 on chromosome 6 (*p* = 4.30 × 10^−6^*)*, were detected. For chest width, SNP s08490.1 on chromosome 5 (*p* = 1.47 × 10^−6^) showed a suggestive association.

Additional suggestive associations were found for oblique length with SNP s22731.1 on chromosome 14 (*p* = 9.31 × 10^−6^), hip width with SNP OAR9_18087803.1 on chromosome 9 (*p* = 9.48 × 10^−6^), and cannon bone circumference with SNP OAR10_1168444.1 on chromosome 10 (*p* = 2.57 × 10^−6^). One SNP, s58409.1 on chromosome 8, was associated with both rump height (*p* = 1.35 × 10^−6^) and withers height (*p* = 1.90 × 10^−7^), suggesting a possible shared genetic basis between these traits.

Annotation results identified several loci that were located near the NIMA-related kinase (*NEK1*), versican (*VCAN*), Erb-B2 receptor tyrosine kinase 4 (*ERBB4*), RUNX1 partner transcriptional co-repressor 1 (*RUNX1T1*), platelet derived growth factor D (*PDGFD*), and neuregulin 1 (*NRG1*) genes. Although the associated SNPs were below the suggestive threshold, these genes have known or plausible roles in skeletal growth, connective tissue organization, and muscle development.

Further research, including functional validation of these candidate genes and replication in larger and independent populations, will be necessary to confirm their effects and assess their potential value for marker-assisted selection in sheep breeding programs.

### 3.3. The KEGG Pathway and GO Enrichment Analyses

The functional enrichment analyses indicated that the candidate genes are primarily involved in key signaling and metabolic pathways related to growth and development. The KEGG pathway enrichment analysis exhibited that *ERBB4* and *NRG1* genes are linked to the ErbB signaling pathway, which plays a role in cell growth and proliferation, while *PDGFD* and *NRG1* are involved in EGFR-tyrosine kinase inhibitor resistance signaling (see Table S2). Overall, these findings suggest that these genes have potential influence over cell survival, proliferation, differentiation, and metabolic activity relevant to growth traits.

**Figure 5:**
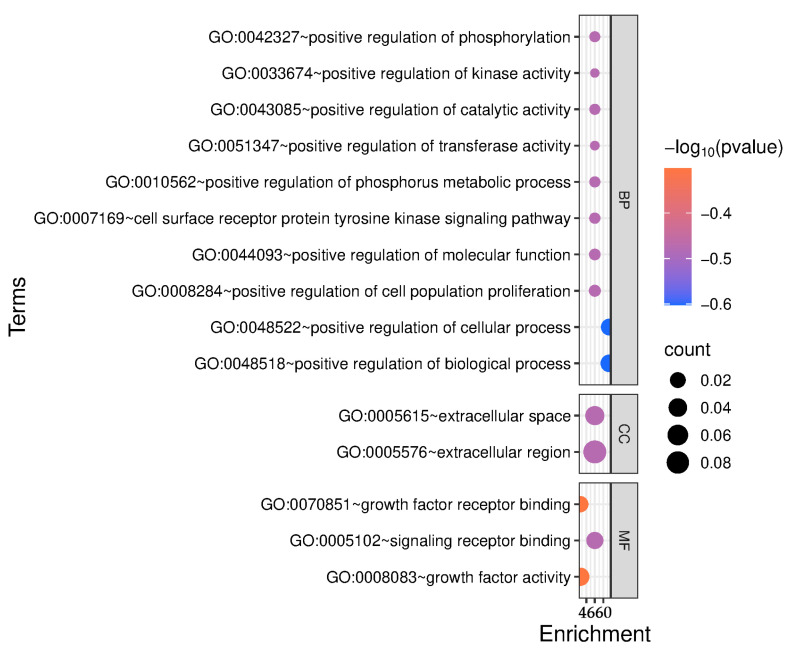
The GO enrichment analysis of candidate genes.

Meanwhile, GO enrichment analysis revealed that the candidate genes are predominantly associated with biological processes related to growth regulation, including the positive regulation of phosphorylation, kinase activity, catalytic activity, and cell population proliferation (Figure 5). Notably, several genes were enriched in the cell surface-receptor protein tyrosine kinase signaling pathway, highlighting their roles in key signaling mechanisms. The cellular component terms indicated that many gene products function in the extracellular space, suggesting involvement in intercellular communication. Furthermore, molecular function enrichment included growth factor activity and receptor binding, supporting the hypothesis that these genes play critical roles in growth factor-mediated signaling. Overall, these findings suggest that the candidate genes contribute to metabolic regulation, signal transduction, and cellular proliferation relevant to growth and development.

## 4. Discussion

In Kazakhstan, there is a notable demand for meat-fat sheep breeds in both domestic and international markets, which has led to the prioritization of certain breeds for breeding purposes. To the best of our knowledge, this study is the first to apply the OvineSNP50 Genotyping BeadChip to Kazakh fat-tailed coarse-wool sheep.

Candidate gene discovery for body confirmation traits in sheep will aid in genetic selection for improved growth and body structure. Multiple SNPs significantly related to growth traits were found through GWAS in German Merino and Qira Black sheep [20]. In our study, SNPs were observed in the *NEK1* gene indicated possible but inconclusive associations with several growth-related traits, including cannon bone circumference, chest depth, chest width, rump height, and withers height. The *NEK1* gene encodes a serine/threonine kinase that plays key roles in several cellular processes, such as cell cycle regulation, DNA damage response and repair, apoptosis, and cell survival [21]. Loss of *NEK1* function impairs primary cilia formation [22]. Given that primary cilia regulate key signaling pathways involved in adipogenesis, *NEK1* function may play a role in adipogenesis by disrupting ciliogenesis.

In sheep breeding programs, live weight is often used as a primary trait to assess genetic potential for growth and meat production. In a previous study, several candidate genes (e.g., *NCAPG*, *MACF1*, *ANKRD44*, *SYN3*, *FUK)* were identified via GWAS as being associated with live weigh in Alpine sheep [23]. Notably, the current study detected a SNP within the *VCAN* gene related to live weight, although it did not reach the genome-wide or suggestive significance thresholds. The *VCAN* gene encodes a large extracellular matrix (ECM) proteoglycan that plays a key role in cell adhesion, proliferation, and migration [24]. Versican is particularly abundant in developing tissues, including cartilage, connective tissue, skin, and is involved in modulating the ECM during growth and remodeling [25]. High-fecundity sheep exhibited RNA editing sites (RESs) involving the *VCAN* gene during the luteal phase, suggesting its potential role in reproductive tissue remodeling and fecundity regulation [26]. However, no correlation between this gene and growth traits in livestock has been reported to date.

In this study, the SNP in *PDGFD* was related to cannon bone circumference in the studied sheep population. Multiple studies have identified *PDGFD* as a key gene underlying the fat-tail phenotype in sheep [27,28]. Also, 18 bp insertion/deletion site was related to sheep litter size [29]. Genotype–phenotype association study showed that a distinct short variation in this gene was correlated with several body measurement traits [30]. Another member of this family PDGFB activate the PDGF receptor β (PDGFRβ) by promoting preadipocyte proliferation, contributing to adipose tissue expansion, and is also involved in the regulation of glucose metabolism [31]. These findings suggest a potential functional role of this gene in body conformation traits, warranting further investigation.

Furthermore, the SNP that demonstrated a weak association with hip width was located near to the *RUNX1T1* gene that encodes a member of the myeloid translocation gene (MTG) family of transcriptional co-repressors. These proteins regulate gene expression by interacting with transcription factors and recruiting histone deacetylases, thereby playing crucial roles in cell differentiation and proliferation [32]. Knockdown of the long splice variant of *RUNX1T1* (*RUNX1T1-L*) in ovine preadipocytes enhanced their differentiation and promoted lipid accumulation, suggesting a regulatory role in adipogenesis [33]. Prior findings indicate that *RUNX1T1* acts as a novel regulator of myogenic differentiation by modulating the calcium signaling pathway, highlighting its potential role in muscle development [34]. Given that *RUNX1T1* was associated with hip width, a trait often influenced by adipose distribution, it may also be involved in fat deposition in the tail, suggesting a broader role in regulating body fat distribution in sheep.

The *NRG1* gene encodes a member of the neuregulin family of proteins, which are key signaling molecules involved in neuronal development and function [35]. NRG1 exerts its effects by binding to ErbB receptors, activating pathways like PI3K-Akt-mTOR and Jak-STAT pathways, which regulate cell growth and differentiation [36]. Notably, it was revealed that *NRG1* regulates adipose differentiation in subcutaneous human stem cells, with its expression influenced by DNA methylation [37]. Furthermore, *ERBB4* is part of the ErbB receptor family, that is activated by *NRG1*, that activates pathways like PI3K–Akt, which is crucial for cell proliferation, differentiation, and tissue development [38]. A previous study using omics approaches reported an association between the *ERBB4* gene and hyperpigmentation in sheep [39]. Although direct evidence is lacking, *NRG1* and *ERBB4* may influence growth traits in sheep by modulating adipocyte development, potentially playing an indirect yet significant role in shaping body conformation traits.

The *ADAM12* gene that encodes a membrane-anchored protein that is part of the ADAM family, was associated with chest depth in our study. *ADAM12* is involved in insulin-like growth factor (IGF) signaling, influencing preadipocyte cell proliferation and differentiation [40]. In addition, it has been shown that transgenic mice expressing ADAM12-S display enhanced longitudinal bone growth [41]. A GWAS in cattle identified a novel mutation in the *ADAM12* gene associated with muscle development and body size [42]. Together, these findings suggest that *ADAM12* may play a crucial role in regulating growth traits in Kazakh fat-tailed coarse-wool sheep and warrants further investigation to clarify its functional significance in this breed.

The KEGG and GO enrichment analyses indicated that the candidate genes are primarily involved in key signaling pathways, including ErbB and EGFR signaling, and biological processes such as phosphorylation, kinase activity, and cell proliferation. Previous study identified phosphorylation and kinase activity as significant terms for productivity traits in sheep [43]. EGFR plays a critical role in bone development by regulating osteoblast maturation and inhibiting the mTOR pathway to control ossification [44]. These findings suggest that these genes may influence growth traits by regulating cellular signaling, energy metabolism, and tissue development.

The scarcity of genome-wide significant SNPs observed in our study likely underscores the complex nature and analytical challenges of dissecting quantitative traits. While the present GWAS identified a few suggestive loci associated with body conformation traits in Kazakh fat-tailed coarse-wool sheep, future studies would benefit from larger sample sizes to improve statistical power and validation.

## 5. Conclusions

This study identified several genomic loci and candidate genes, such as *VCAN*, *NEK1*, *NRG1*, *ADAM12*, *ERBB4*, *RUNX1T1*, *PDGFD* related to body conformation traits in Kazakh fat-tailed coarse-wool sheep. These findings provide a foundation for marker-assisted selection to improve growth performance, and future work should focus on functional validation of the identified genes.

## Figures and Tables

**Table 1:** Descriptive statistics for phenotype values for each trait.

Traits	Mean ± SD	CV (%)	Min	Max
Live weight, (kg)	63.1 ± 3.9	6.2	52	73
Withers height, (cm)	74.2 ± 3.6	4.8	63	82
Rump height, (cm)	75.7 ± 3.5	4.6	65	83
Chest width, (cm)	25.7 ± 2.1	8.1	21	34
Chest depth, (cm)	35.4 ± 1.7	4.9	29	40
Chest girth, (cm)	101.5 ± 4.3	4.2	86	116
Oblique length, (cm)	64.5 ± 3.4	5.3	55	75
Hip width, (cm)	20.1 ± 2	9.8	15	25
Cannon bone circumference, (cm)	9.2 ± 1	10.8	8	12

Note: sample size, *n* = 295 sheep; CV, coefficient of variation; SD, standard deviation.

## Data Availability

The data available upon request.

## Supplementary Materials

The following supporting information can be downloaded at: https://www.mdpi.com/article/10.3390/genes16091023/s1, Figure S1: The quantile–quantile (QQ) plots of traits. Table S1: SNPs and candidate genes revealed by GWAS. Table S2: The KEGG pathway enrichment analysis of candidate genes.

## Abbreviations

The following abbreviations are used in this manuscript:

*ADAM12*	ADAM metallopeptidase domain 12
*ADGRL3*	Adhesion G protein-coupled receptor L3
*ANKRD44*	Ankyrin repeat domain 44
*CALP*	Calpastatin
*FGF12*	Fibrolast growth factor 12
*FTO*	FTO alpha-ketoglutarate dependent dioxygenase
*ELOVL2*	ELOVL fatty acid elongase 2
*ERBB4*	Erb-B2 receptor tyrosine kinase 4
*GWAS*	Genome-wide association study
*HOXB13*	Homeobox B13
*IGF-1*	Insulin-like growth factor 1
*IGFBP6*	Insulin-like growth factor binding protein 6
*MAS*	Marker-assisted selection
*MACF1*	Microtubule Actin Crosslinking Factor 1
*MSTN*	Myostatin
*NCAPG*	Non-SMC condensin I complex subunit G
*NEK1*	NIMA-related kinase 1
*NRG1*	Neuregulin 1
*PDGFD*	Platelet-derived growth factor D
*RUNX1T1*	RUNX1 partner transcriptional co-repressor 1
*SNP*	Single-nucleotide polymorphisms
*VCAN*	Versican
