# Supplementary material for: Genome-Wide Association Study for Body Conformation Traits in Kazakh Fat-Tailed Coarse-Wool Sheep
Source: Genes (Basel). 2025 Aug 29;16(9):1023. doi: 10.3390/genes16091023 (PMC12469880; doi:10.3390/genes16091023)
Supplement: Supplementary file 1 [file genes-16-01023-s001.zip › Supplementary material.pdf]

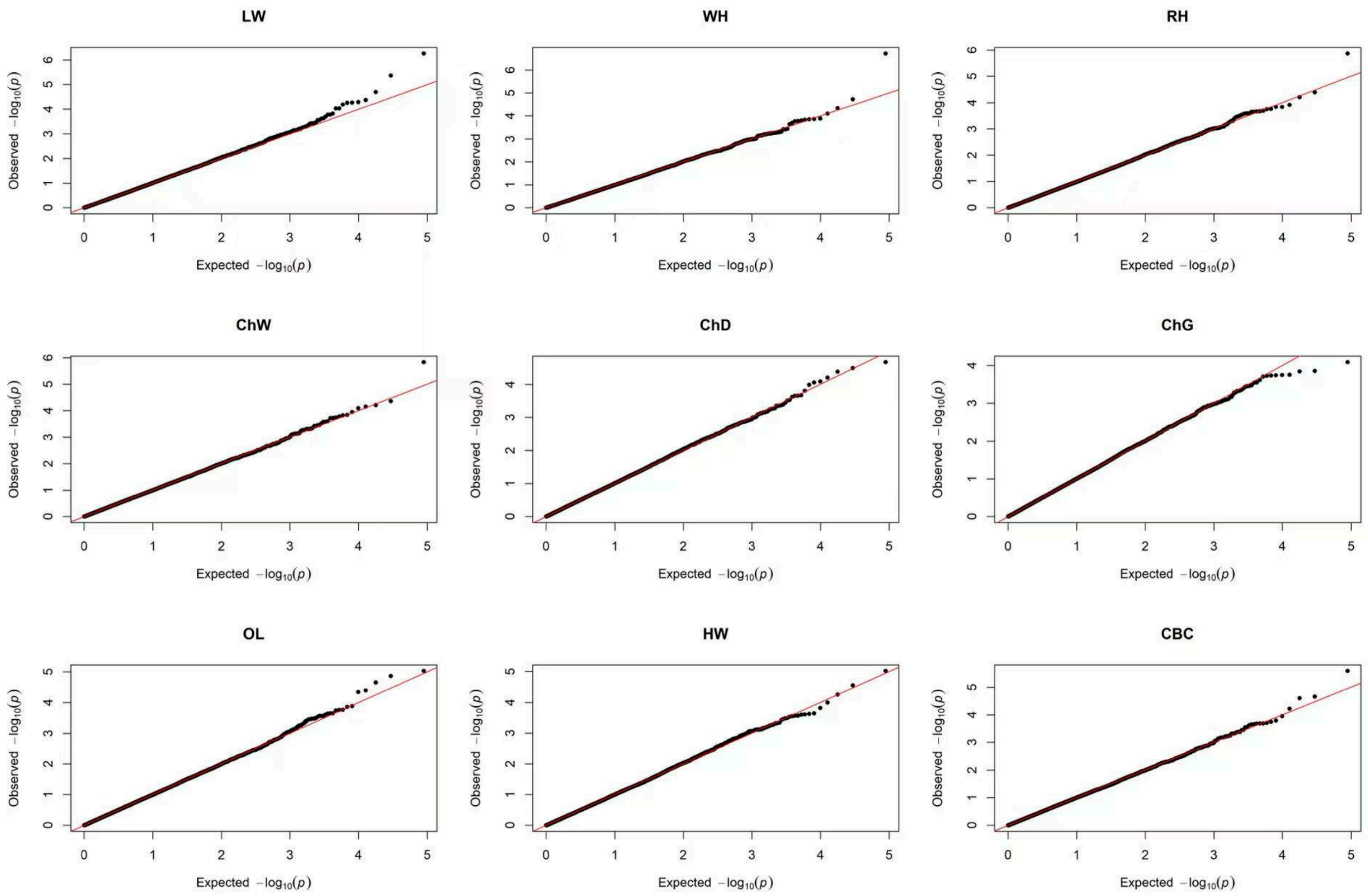

Figure S1. The quantile-quantile (QQ) plots of traits. LW, live weight; WH, withers height; RH, rump height; ChW, chest width; ChD, chest depth; ChG, chest girth; OL, oblique length; HW, hip width; CBC, cannon bone circumference.
